# Integron, Plasmid and Host Strain Characteristics of *Escherichia coli* from Humans and Food Included in the Norwegian Antimicrobial Resistance Monitoring Programs

**DOI:** 10.1371/journal.pone.0128797

**Published:** 2015-06-05

**Authors:** Marianne Sunde, Gunnar Skov Simonsen, Jannice Schau Slettemeås, Inger Böckerman, Madelaine Norström

**Affiliations:** 1 Department of Laboratory Services, Norwegian Veterinary Institute, Oslo, Norway; 2 Department of Microbiology and Infection Control, University Hospital of North Norway, Tromsø, Norway; 3 Research Group for Host-Microbe Interaction, Institute of Medical Biology, Faculty of Health Sciences, University of Tromsø —The Norwegian Arctic University, Tromsø, Norway; 4 Department of Health Surveillance, Norwegian Veterinary Institute, Oslo, Norway; University of Manchester, UNITED KINGDOM

## Abstract

Antimicrobial resistant *Escherichia coli* (n=331) isolates from humans with bloodstream infections were investigated for the presence of class 1 and class 2 integrons. The integron cassettes arrays were characterized and the findings were compared with data from similar investigations on resistant *E*. *coli* from meat and meat products (n=241) produced during the same time period. All isolates were obtained from the Norwegian monitoring programs for antimicrobial resistance in human pathogens and in the veterinary sector. Methods used included PCR, sequencing, conjugation experiments, plasmid replicon typing and subtyping, pulsed-field-gel-electrophoresis and serotyping. Integrons of class 1 and 2 occurred significantly more frequently among human isolates; 45.4% (95% CI: 39.9-50.9) than among isolates from meat; 18% (95% CI: 13.2 -23.3), (*p*<0.01, Chi-square test). Identical cassette arrays including *dfrA1-aadA1*, *aadA1*, *dfrA12*-orfF-*aadA2*, *oxa-30-aadA1* (class 1 integrons) and *dfrA1-sat1-aadA1 *(class 2 integrons) were detected from both humans and meat. However, the most prevalent cassette array in human isolates, *dfrA17-aadA5*, did not occur in isolates from meat, suggesting a possible linkage between this class 1 integron and a subpopulation of *E*. *coli* adapted to a human host. The *drfA1-aadA1 *and *aadA1 *class 1 integrons were found frequently in both human and meat isolates. These isolates were subjected to further studies to investigate similarities with regard to transferability, plasmid and host strain characteristics. We detected incF plasmids with pMLST profile F24:A-:B1 carrying *drfA1-aadA1 *integrons in isolates from pork and in a more distantly related *E*. *coli* strain from a human with septicaemia. Furthermore, we showed that most of the class 1 integrons with *aadA1* were located on incF plasmids with pMLST profile F51:A-:B10 in human isolates. The plasmid was present in unrelated as well as closely related host strains, demonstrating that dissemination of this integron also could be attributed to clonal spread. In conclusion, among the systematically collected isolates from two different sources, some significant differences concerning integron prevalence and integron variants were observed. However, closely related plasmids as vehicles for specific class 1 integrons in isolates from meat and from a human with bloodstream infection were found. The occurrence of similar multi-resistance plasmids in bacteria from a food source and from a human clinical sample highlights the possible role of meat as a source of resistance elements for pathogenic bacteria.

## Introduction

Monitoring programs for antimicrobial resistance in bacteria have been established in many countries during the last 15 years. Data generated by these programs are important for estimation of resistance prevalence for various bacterial species, can be used to follow trends over time, and are important for risk analysis and interventions in order to control the spread of resistant bacteria. The strain collections obtained in monitoring programs represent unique materials for further studies as the isolates have been systematically collected over years, originate from a variety of different sources and represent the national resistance situation in the respective countries. The Norwegian monitoring programs for antimicrobial resistance; NORM for human pathogens and NORM-VET for bacteria of animal origin, have been running for nearly 15 years. Together, these programs collect resistance data from human pathogens, from zoonotic bacteria, from clinical isolates from animals, as well as from indicator bacteria from healthy production animals and domestically produced meat. All isolates obtained in NORM and NORM-VET are stored and frozen for possible future research.

The genetic background for resistance in *Escherichia coli* originating from Norwegian produced poultry, pork, beef (minced meat) and mutton in the NORM-VET program (2000 to 2003) has been previously studied [1,2]. The integron structures appearing in resistant isolates were investigated in detail [1]. Multi-resistance integrons are of great importance in the epidemiology of antimicrobial resistance among Gram-negative bacteria [3,4]. Integrons can incorporate mobile gene cassettes by site-specific recombination [5]. Resistance to important antimicrobial agents used in both human and veterinary medicine can be conferred via gene cassettes within integrons [4,6]. Integrons can also contain genes encoding metallo-beta-lactamases highlighting [7] their role in the dissemination of resistance to last line antimicrobial agents. Although integron gene cassettes are mobile structures, many combinations of gene cassettes seem to be more or less stable like the *dfrA17-aadA5*, *dfrA1-aadA1*and *dfrA12-orfF-aadA2* combinations within class 1 integrons and the *dfrA1-sat1-aadA1* array in class 2 integrons. These arrays have been described from numerous other studies over the last 20 years [8–13]. One reason for their wide dissemination could be that they are part of integrons borne on specific transposons, plasmids or “antibiotic resistance islands” [3,4]. In our previous studies of integron structures in *E*. *coli* from Norwegian produced meat we found that transfer of resistance determinants occurred significantly more often from resistant isolates containing a class 1 integron compared to those without [2]. It is possible that many plasmids carrying class 1 integrons are highly transmissible, with a broad host range allowing them to disseminate throughout different bacterial populations. This may include the more pathogenic bacterial variants associated with severe infections in humans, like those causing blood stream infections (BSI).

*E*. *coli* causing blood stream infection are usually not random isolates from the intestinal flora of the host, but a subset of *E*. *coli* exhibiting a higher level of virulence [14]. Such *E*. *coli* can also be defined as extra-intestinal *E*. *coli* (ExPEC) and can usually be grouped into the phylogenetic group B2 and to some extent group D. The contamination flora of meat and meat products usually contain *E*. *coli* belonging to phylogenetic groups other than B2 [15,16]. Class 1 integrons in *E*. *coli* from the contamination flora of meat may theoretically serve as a reservoir of resistance elements for bacteria causing human disease if the genetic structures are able to intermingle between different compartments of the *E*. *coli* population.

The aims of this study were to estimate the prevalence of class 1 and class 2 integrons, and to characterize cassette content of integrons in resistant *E*. *coli* from humans with bloodstream infections in Norway. A further aim was to compare the findings with “integron profiles” of *E*. *coli* occurring in domestically produced meat from the same time period and to determine if common resistance elements, i. e. transmissible plasmids with class 1 integrons, are shared among food commensals and human pathogens.

## Materials and Methods

### Bacterial isolates

A total of 331 *E*. *coli* isolates from humans with BSI and 241 *E*. *coli* isolates from Norwegian produced meat (cattle, mutton, pork and poultry) were included in this study. All isolates included were classified as resistant to one or more antibacterial agents tested for in the Norwegian monitoring programs for antimicrobial resistance NORM/NORM-VET [17–20]. The 331 human isolates comprised all resistant *E*. *coli* bloodstream isolates during year 2003 except 13 isolates from one laboratory which could not be retrieved due to discontinuation of laboratory services. The isolates from meat originated from samples obtained through representative random sampling schemes. In short, the numbers of samples were proportional to the slaughter volume at the abattoirs the previous year. Slaughter plants representing < = 1% of the slaughter volume were not included. For the year 2003, the sampling was instead performed at 16 cutting plants, each collecting 10 samples. All sampling was performed throughout the year. The isolates were susceptibility tested to a panel of antibiotics during 2000–2003 by the use of a microdilution method (VetMIC, SVA. Uppsala, Sweden).

### Screening for integrons and characterization of genes cassettes

Integron detection and characterization was carried out in the following way: The isolates were screened for the integrase genes of class 1, 2 and 3 integrons using PCR followed by restriction endonuclease digestion as previously described [21]. Strains containing the *intI* and/or the *intI2* genes were subsequently subjected to PCR amplification of the variable regions of class 1 and/or class 2 integrons, respectively. The primers and PCR conditions have been described previously [21,22]. All templates were prepared by the boil lysis method and primer sequences are listed in Table 1.

**Table 1:** Primers used in the PCR experiments.

Primer name	Sequence (5’ → 3’)	Target gene(s) or region	PCR productsize (bp)
hep35	TGC GGG TYA ARG ATB TKG ATT T	*intI1*, *intI2*, *intI3*	491
hep36	CAR CAC ATG CGT RTA RAT	*intI1*, *intI2*, *intI3*	
5’-CS	GGC ATC CAA GCA GCA AG	class 1 integron	Variable
3’-CS	AAG CAG ACT TGA CCT GA	variable region	
hep74	CGG GAT CCC GGA CGG CAT GCA CGA TTT GTA	class 2 integron	Variable
hep51	GAT GCC ATC GCA AGT ACG AG	variable region	
aadaI*	TCG GCG CGA TTT TGC CGG TTA C	*aadA*	

*the aadaI primer was used in combination with the 5’-CS primer for amplification of class 1 integrons lacking the 3’-conserved segment. The other primers were used pairwise as listed.

PCR products generated with primers for amplification of cassette areas were sequenced in order to determine the content and order of inserted cassettes. However, the variable regions of class 1 integrons with *dfrA1*-*aadA1* or *aadA1* as the sole cassette, as well as class 2 integrons with *dfrA1*-*sat1*-*aadA1*, were characterized using PCR and subsequent restriction endonuclease digestion as previously described [1].

The usual structure of class 1 integrons consists of two conserved segments flanking the cassette area. The 5’-conserved segment contains *intI* and the insertion site for gene cassettes, *attI*. The 3’-conserved segment contains a truncated version of *qacE* and the sulphonamide resistance gene *sul1*. The 3’ conserved segment of some class 1 integrons may be absent resulting in lack of the hybridization site of the 3’ conserved segment specific primer. Combination of an *aadA* reverse primer (aadaI) with an *intI* primer (5’-CS) enabled us to amplify cassette areas in class 1 integrons lacking the 3’ conserved segment, but containing an *aadA*-like cassette. Primer sequences, annealing temperatures and PCR conditions have been described previously [1]. The amplicons generated were subsequently verified by sequencing. Positive and negative controls were included in all PCR reactions. *E*. *coli* Se 131 (Acc no AJ238350) containing *dfrA1*-*aadA1* within a class 1 integron, was used as positive control for detection of *intI1* and the cassette area of class 1 integrons *E*. *coli* U56 containing Tn*7*, was used as positive control for detection of *intI2* and the cassette array *dfrA1*-*sat1*-*aadA1* of class 2 integrons.

### Conjugation experiments

Conjugation was carried out in broth with *E*. *coli* DHα as recipient, as previously described [2]. *E*. *coli* resistant to quinolones, but susceptible to kanamycin was conjugated with *E*. *coli* One Shot cells containing the pCRII vector encoding kanamycin resistance (Invitrogen, LifeTechnologies, Thermo Fisher Scientific Inc., Waltham, MA, USA) as recipient. Transconjugants were selected as previously described [2] using Mueller-Hinton agar plates containing 50 μg/ml nalidixic acid (*E*. *coli* DH5α) or 50 μg/ml kanamycin (One Shot *E*. *coli*). Transconjugants were subjected to PCR to verify the presence of integrase genes, and they were also plated out on lactose-saccharose-bromthymol blue agar plates in parallel with the donor strain for visual inspections of colony morphology to confirm the transconjugants as real transconjugants and not donor strains with mutations leading to a resistant phenotype. There are notable differences in colony morphology between the recipient and donor strains.

### Plasmid characterization

A subset of transconjugants was subjected to PCR based replicon typing (PBRT) as previously described [23]. The template used was DNA extracted by NucliSENS easyMag extractor (bioMérieux, Marcy l’Etoile, France). Positive and negative controls were included in each run. Subtyping of incF plasmids was carried out by the use of plasmid multilocus sequence typing (pMLST) method as described by Villa et al. [24]. Plasmid content and plasmid sizes were determined by S1 digestion (Invitrogen) of agarose-embedded DNA and pulsed-field gel electrophoresis (PFGE) as described previously [25].

### Determination of phylogroups

Phylogenetic background was determined by the use of multiplex PCR as previously described [26]. The amplicons from one strain (2003500827) belonging to the B2 group yielding amplicons for all four primer pairs, were sequenced and confirmed for quality assurance of the method.

### Pulsed-field gel electrophoresis (PFGE)

PFGE was performed as described for *E*. *coli* using the protocol recommended by PulseNet [27] with the following minor modifications: the gel running time was 24 hours and the temperature was 12°C. The restriction enzyme used was *Xba*I (Sigma, St Louis MO). The banding patterns were evaluated by using a combination of visual inspection and the Bionumerics software (Bionumerics, Applied Maths, Kortrijk, Belgium).

### Serotyping

Serotyping (O, K and H antigens) was carried out by Statens Serum Institut (SSI) in Denmark (isolate no 2003500827). The O6 antigen was detected by PCR with primers described by Li et al. [28]. The primer sequences are listed in Table 1. Isolate number 2003500827 was included as positive control (typed to *E*. *coli* O6:K5:H1 by SSI, Copenhagen, Denmark).

### DNA sequencing

Nucleotide sequences were determined using the BigDye Terminator v3.1/1.1 Cycle Sequencing kit (Applied Biosystems, Life Technologies, Thermo Fisher Scientific Inc., Waltham, MA). Sequencing reactions were run on a capillary sequencer 3130*xl* Genetic Analyzer (Applied Biosystems) and sequences were analyzed using the BioEdit program (www.mbio.ncsu.edu/BioEdit/bioedit.html), the CLC bio Combined Workbench (CLC bio A/S, Aarhus, Denmark), and the NCBI GeneBlast2 and ClustalW programs via the internet.

### Statistical analysis

Statistical analyses were performed with the software package R v 2.15.1 [29]. Comparison of proportions of integrons in human isolates versus meat isolates was assessed using the Chi square test.

## Results

### Prevalence of integrons and gene cassette arrays

Integrons of class 1 or class 2 were detected in 45.4% (95% CI: 39.6–51.1) of the 331 resistant *E*. *coli* isolates from humans with bloodstream infections. Class 1 integrons occurred in 39% (95% CI: 33.7–44.5) and class 2 integrons were detected in 7.3% (95% CI: 4.7–10.6) of the isolates. Three isolates contained both a class 1 and a class 2 integron. Integrons occurred significantly more frequently among human isolates (45.4% (95% CI: 39.9–50.9) than among isolates from meat 18% (95% CI: 13.2–23.3) (p<0.01, Chi-square test). Table 2 gives an overview of integrons detected and gene cassettes inserted within them. Integrons and gene cassettes in resistant *E*. *coli* from meat produced in Norway are given in the same table.

**Table 2:** Number of integrons and different cassette arrays occurring in antimicrobial resistant *Escherichia coli* originating from humans with blood stream infections and in antimicrobial resistant *E*. *coli* originating from meat and meat products.

Integrons and cassette arrays	Human clinical isolates(n = 331)	Isolates from meat (n = 241)^c^
Class 1 integrons:		
*dfrA1-aadA1*	17	12
*aadA1*	16	4
*dfrA17-aadA5*	39	0
*dfrA5*	10	0
*dfrA7*	6	0
*dfrA12-orfF-aadA2* ^a^ ^,^ ^c^	2	3
*oxa-30-aadA1a*	4	1
*dfrA12-orfF-aadA2*	1	0
*aacA4*, *catB3*, *dfrA1*	1	0
*dfrA16-aadA1*	1	0
*dfrA5- aadA5*	1	0
Undetermined^a^	28	7
*dfrIIa-aadA1-catB2*	0	2
**Total class 1 integrons:**	**126**	**29**
Class 2 integrons:		
*dfrA1-sat1-aadA1*	18	12
*estX-sat2-aadA1*	1	0
*dfrA1-sat1*	1	0
*dfrA1* ^b^	1	0
*sat-sat1-aadA1*	0	2
**Total class 2 integrons:**	**21**	**14**
Class 1 and class 2 integrons in the same isolate:Class 1 integron (*aadA1*) +Class 2 integron (*dfrA1-sat1-aadA1*)Class 1 integron (*aadA1*) +Class 2 integron (*dfrA1-sat1*)	21	00
**Total class 1 + class 2 integrons in the same isolate:**	**3**	**0**

^a^Class 1 integron lacking the 3- conserved segment

^b^One *dfrA1* cassette is located as the first cassette, the remaining cassettes (if any) are not characterized

^c^ The integrons in *E*. *coli* from meat and in one human strain (with *dfrA12-orfF-aadA2* cassettes) have been characterized previously [1,30].

The most frequent cassette array in class 1 integrons from human pathogens, *dfrA17-aadA5*, did not occur in *E*. *coli* from meat, whereas the most common cassette array in class 1 integrons in *E*. *coli* from meat, *drfA1-aadA1*, occurred as the second most common cassette structure in human isolates (Table 2). Other cassettes/cassette combinations were present in *E*. *coli* of both categories as seen for *aadA1*, *dfrA12-orfF-aadA2*, *oxa-30-aadA1* in class 1 integrons and *dfrA1-sat1-aadA1* in class 2 integrons (Table 2). Some of the cassettes were only observed in isolates from humans like *dfrA5* and *dfrA7* (as the only cassette) in ten and six isolates, respectively, whereas the cassette array *dfraIIa-aadA1-catB2* only occurred in isolates from meat (n = 2).

Class 1 integrons may occasionally lack the usual 3’ conserved segment [1,30]. Attempts were made to amplify and partly characterize the variable region of such integrons, by combining primers for the integrase (*intI*) and an *aadA* cassette. The *aadA* cassette is one of the most frequent cassettes in integrons, and it was therefore chosen as target DNA in an attempt to amplify the cassette region. However, no PCR products were obtained except for two isolates, with the *dfrA12-orfF-aadA2* array (Table 2).

The most commonly identified class 1 integrons that were present in both categories of isolates were *drfA1-aadA1* and *aadA1* (Table 2). These isolates were selected for further studies in order to determine possible similarities with regard to transferability of integrons, plasmids involved and host strain characteristics.

### Class 1 integrons with the *drfA1-aadA1* cassette array

Phylogenetic typing of isolates with this particular integron showed that the BSI isolates belonged to the A, B2, and D groups, whereas the meat isolates grouped into A, B1 and D (Tables 3 and 4). Ten of the 12 isolates from meat carried the *drfA1-aadA1* integron on a self-transmissible plasmid (Table 4). Among the BSI isolates, seven out of 17 isolates were not able to transfer the integron structure when conjugation was performed (Table 3). Replicon typing of transconjugants showed that incF plasmids dominated among the isolates from meat. The conjugative plasmids in four of the human isolates also belonged to incF as shown in Table 3. However, the integron structure was also associated with conjugative plasmids of other incompatibility groups in isolates from humans (Table 3). Some of the tranconjugants contained more than one replicon and the location of the integron on a plasmid of a specific incompatibility group could therefore not be determined.

**Table 3:** Antimicrobial resistant *Escherichia coli* isolates containing class 1 integrons with the *dfrA1-aadA1*cassette array.

Isolate id	Hospital	Phylogenetic group	Cassette content	Conjugation^a^	PBRT^b^	incF subtyping^c^	PFGE
14141344	Ullevål	B2	*dfrA1-aadA1*	+	FIA,Frep	F-:A1:B-	-
200330776	Tønsberg	B2	*dfrA1-aadA1*	+	FIB	F-:A-:B1	-
200330220	Levanger	B2	*dfrA1-aadA1*	+	FIB,Frep	F24:A-:B1	ut
2003303565	Radiumhosp	A	*dfrA1-aadA1*	+	Frep,BO	F2:A-:B-	-
S10	Stavanger	D	*dfrA1-aadA1*	+	N	-	-
14143751	Ullevål	B2	*dfrA1-aadA1*	+	BO	-	-
200361798	St Olavs	D	*dfrA1-aadA1*	+	BO	-	-
200343196	Lillehammer	A	*dfrA1-aadA1*	+	B/O, I,P	-	-
200340328	Lillehammer	D	*dfrA1-aadA1*	+	B/O, I,P	-	ut
2003304240	Radiumhosp	D	*dfrA1-aadA1*	+	B/O, I,P	-	ut
20035877	Levanger	B2	*dfrA1-aadA1*	÷	-	-	-
200334890	Tromsø	A	*dfrA1-aadA1*	÷	-	-	-
20035413	Tromsø	A	*dfrA1-aadA1*	÷	-	-	-
200325934	Tromsø	A	*dfrA1-aadA1*	÷	-	-	-
200327757	Tromsø	A	*dfrA1-aadA1*	÷	-	-	-
200353155	Lillehammer	A	*dfrA1-aadA1*	÷	-	-	-
200353153	Lillehammer	A	*dfrA1-aadA1*	÷	-	-	-

The isolates were recovered from humans with blood stream infections.

^a^Transferability of plasmids with class 1 integron by conjugation

^b^PBRT = Plasmid-based replicontyping (plasmid replicon(s) in transconjugant); + = transconjugants

were obtained, ÷ = no transconjugantes were obtained

^c^Subtyping of conjugative incF plasmid with class 1 integron

ut = unique type, one isolate with this banding pattern was found

**Table 4:** Antimicrobial resistant *Escherichia coli* isolates containing class 1 integrons with the *dfrA1-aadA1* cassette array.

Isolate id	Type of meat, isolated year	Phylogenetic group	Cassette content	Conjugation^a^	PBRT^b^	incF subtyping^c^	PFGE
S4	Pork, 2001	B1	*dfrA1-aadA1*	+	FIB,Frep	F24:A-:B1	a
S5	Pork, 2001	B1	*dfrA1-aadA1*	+	FIB,Frep	F24:A-:B1	a
S357	Pork, 2001	B1	*dfrA1-aadA1*	+	FIB,Frep	F24:A-:B1	a
S373	Pork, 2001	B1	*dfrA1-aadA1*	+	FIB,Frep,I	F24:A-:B1	a
K162	Broiler, 2000	A	*dfrA1-aadA1*	+	FIB,Frep	F76:A-:B1	n-t
K192	Broiler, 2000	A	*dfrA1-aadA1*	+	FIB,Frep	F76:A-:B1	n-t
2002-01-1432-5	Broiler, 2002	B1	*dfrA1-aadA1*	+	FIB,Frep	F24:A-:B6	ut
K31	Broiler, 2000	B1	*dfrA1-aadA1*	+	FIB,Frep	F24:A-:B6	ut
K143	Broiler, 2000	D	*dfrA1-aadA1*	+	FIB,Frep,I.P	F34:A-:B1	-
K144	Broiler, 2000	D	*dfrA1-aadA1*	+	FIB, I,P	F-:A-:B1	-
K136	Broiler, 2000	A	*dfrA1-aadA1*	÷	-	-	-
S376	Pork, 2001	B1	*dfrA1-aadA1*	÷	-	-	-

The isolates were recovered from Norwegian produced meat and meat products.

^a^Transferability of plasmids with class 1 integron by conjugation; + = transconjugants were obtained, ÷ = no transconjugantes were obtained

^b^PBRT = Plasmid-based replicontyping (plasmid replicon (s) in transconjugant)

^c^Subtyping of conjugative incF plasmid with class 1 integron

n-t = non-typeable

ut = unique type, one isolate with this banding pattern was found

pMLST of the incF plasmids showed that none of the incF plasmids in isolates of human origin had the same pMLST type when compared with each other. In isolates from meat, however, a major part of the plasmids belonged to the FIB incompatibility group and had FAB formulas with relatively few differences, thus suggesting circulation of related plasmids carrying integrons with the *drfA1-aadA1* cassettes in *E*. *coli* from pork and poultry (Table 4). All these isolates also carried the *tetA* tetracycline resistance determinant (data not shown). Comparison between plasmids of meat and human origin revealed that four isolates from pork and one isolate from a human contained an incF plasmid with the same FAB formula; F24:A-:B1. This indicates the presence of closely related multi-resistance plasmids in *E*. *coli* isolates from pork and in one clinical isolate of human origin. The isolates from swine belonged to the phylogenetic group B1 whereas the human isolate belonged to the B2 group. PFGE showed that three of the isolates from pork produced identical PFGE banding patterns and that the remaining produced a pattern with only two band difference. The human isolate produced a distinct banding pattern, demonstrating the presence of the plasmid in unrelated host strains in isolates of human and meat origin. Plasmids of approximately 160 kb were present in all isolates from pork and also in the human isolate. The isolates from pork were collected from meat samples during a four month period suggesting occurrence and persistence of a multi-resistant *E*. *coli* clone in meat of swine during this time period.

### Class 1 integrons with the *aadA1* cassette

All isolates of human origin belonged to the B2 phylogenetic group, except one that belonged to the A group. The isolates from meat grouped into the A, B1 and D groups (Tables 5 and 6). Most of the isolates from humans with this particular integron carried it on a conjugative plasmid (12 out of 16 isolates). Four meat isolates contained integrons with this cassette and only two of them transferred the integron when conjugation was performed (Tables 5 and 6). Replicon typing of the conjugative plasmids from the human isolates revealed an incF plasmid was present in all isolates. The conjugative plasmids in meat isolates were associated with incP and/or inc B/O, further comparisons between human and meat isolates/plasmids were therefore not performed.

**Table 5:** Antimicrobial resistant *Escherichia coli* isolates containing class 1 integrons with an *aadA1* cassette as the only inserted gene cassette.

Isolate id	Hospital	Phylogenetic group	Cassette content	Conjugation^a^	PBRT^b^	incF subtyping^c^	PFGE
14142148	Ullevål	B2	*aadA1*	**+**	FIB,Frep	F51:A-:B10	b
2003614011	Buskerud	B2	*aadA1*	**+**	FIB,Frep	F51:A-:B10	b
200337758	Tønsberg	B2	*aadA1*	**+**	FIB,Frep	F51:A-:B10	b
2003108761	Haugesund	B2	*aadA1*	**+**	FIB,Frep	F51:A-:B10	b
200312531	St Olavs	B2	*aadA1*	**+**	FIB,Frep	F51:A-:B10	b
2003500827	Bærum	B2	*aadA1*	**+**	FIB,Frep	F51:A-:B10	b
2003500361	Bærum	B2	*aadA1*	**+**	FIB,Frep	F51:A-:B10	b
200314544	Rikshospitalet	B2	*aadA1*	**+**	FIB,FrepBO	F51:A-:B10	ut
200320223	Førde	B2	*aadA1*	**+**	FIB,Frep	F51:A-:B10	ut
0327154	Molde	B2	*aadA1*	**+**	FIB,Frep	F51:A-:B10	ut
200333380	Tromsø	B2	*aadA1*	**+**	FIB,Frep	F51:A-:B10	ut
2003635225	Haugesund	B2	*aadA1*	**+**	FIB,Frep	F51:A-:B10	ut
200313666	St Olavs	A	*aadA1*	**÷**	-	**-**	**-**
200334232	Tønsberg	B2	*aadA1*	**÷**	**-**	**-**	**-**
200355042	Lillehammer	B2	*aadA1*	**÷**	**-**	**-**	**-**
310241531	Haukeland	B2	*aadA1*	**÷**	**-**	**-**	**-**

The isolates were recovered from humans with blood stream infections.

^a^Transferability of plasmids with class 1 integron by conjugation; + = transconjugants were obtained, ÷ = no transconjugantes were obtained

^b^PBRT = Plasmid-based replicontyping (plasmid replicon(s) in transconjugant)

^c^Subtyping of conjugative incF plasmid with class 1 integron

ut = unique type, one isolate with this banding pattern was found

**Table 6:** Antimicrobial resistant *Escherichia coli* isolates containing class 1 integrons with an *aadA1* cassette as the only inserted gene cassette.

Isolate id	Type of meat, isolated year	Phylogenetic group	Cassette content	Conjugation^a^	PBRT^b^	incF subtyping	PFGE profile
S-95	Swine, 2000	A	*aadA1*	+	P,B/O	-	-
2002-01-1588-6	Swine, 2002	A	*aadA1*	+	BO	-	-
2002-01-1117-8	Swine, 2002	B1	*aadA1*	÷	-	-	-
K142	Broiler, 2001	D	*aadA1*	÷	-	-	-

The isolates were recovered from Norwegian produced meat.

^a^Transferability of plasmids with class 1 integron by conjugation; + = transconjugants were obtained, ÷ = no transconjugantes were obtained

^b^PBRT = Plasmid-based replicontyping (plasmid replicon(s) in transconjugant)

The incF plasmids in the human isolates all belonged to the same sub-category, with the formula F51:A-:B10. A plasmid of approximately 110 kb was present in all isolates (except one that was not investigated). PFGE was performed to investigate possible clonal relationship between the human isolates harbouring this plasmid. Seven isolates produced banding patterns with more than 85% similarity suggesting a possible clonal distribution of this *E*. *coli* variant. The seven isolates originated from six different hospitals spread across Norway. Furthermore, one representative isolate (2003 3500827) was serotyped as O6:K5:H1 (SSI, Denmark), a serotype known to be involved in bloodstream infections in human. The remaining six closely related isolates were subjected to O6 specific PCR with a positive result. The remaining five isolates containing the particular plasmid produced distinct PFGE patterns indicating presence of the plasmid in unrelated host strains as well (Table 5).

## Discussion

Integrons have been characterized in many studies since their discovery in the late 1980s. However, most of the isolates in previous studies have not been collected in a randomized manner, but rather based on convenience sampling or availability of strains, thus making generalization difficult with regard to prevalence of integron structures and occurrence of cassette variants within them. The *E*. *coli* strains included in this study were collected through the Norwegian national resistance monitoring programs. Consecutive human *E*. *coli* bloodstream isolates from all clinical laboratories are included in NORM. Each patient is represented by a single isolate for each infectious episode to preclude repetitive inclusion. The NORM strain collection is thus an unbiased representation of systemic *E*. *coli* isolates on a national level. The meat isolates were retrieved through a randomized sampling scheme, thus representing the *E*. *coli* population in the chosen products. The mapping of integrons in such collections represents a novel approach in describing prevalence and integron characteristics in two different *E*. *coli* populations, collected within the same geographical area and timeframe. In addition, a relatively high number of isolates were investigated.

The present study demonstrates that the most prevalent class 1 integron of human origin, *dfrA17-aadA5* detected in 39 out of 126 isolates, did not occur in isolates from meat. This class 1 integron may be linked to a *E*. *coli* subpopulation which is able to cause severe infection, or highly adapted to colonization of a human host. It is also possible that isolates with this particular integron are genetically related to each other, and the integron associated with certain clonal lineages. A recent study from the US showed that a considerable proportion of investigated *E*. *coli* blood stream isolates grouped into a limited number of sequence types [31]. Other studies reporting class 1 integrons from production animal reservoirs have to a large extent also documented the *dfrA17-aadA5* array to be less prevalent in investigated isolates [8,9,32–36], whereas the integron variant has been reported as common among isolates of human origin in previous studies [8,10,12,13,36].

Conversely, our study also reports closely related incF plasmids as vehicles for specific class 1 integrons in isolates from swine meat and a human with septicaemia. This demonstrates the occurrence of similar multi-resistance plasmids in bacteria from a food source and from a human clinical sample, highlighting the possible role of meat as a source of resistance elements for pathogenic bacteria. A more extensive study is needed to fully evaluate the impact of resistance plasmids in meat on human health. However, a recently published study evaluating the nucleotide sequences of several extended-spectrum-beta-lactamase (ESBL) plasmids from animals and humans in the Netherlands have just shown that highly similar plasmids occurred in both reservoirs [37]. The F24:A-:B1 plasmid may be a successful plasmid with a broad dissemination in the whole *E*. *coli* population, including commensals and pathogens, and the contamination flora of meat may thus not necessarily be the main reservoir. Only a few studies have reported incF plasmid with this pMLST profile, but at least one previous study has documented F24:A-:B1 plasmids in ExPEC strains [24,38].

Many studies have attributed spread of class 1 integrons to their localization on transferable plasmid with a broad host range. We have shown that dissemination of a specific integron can also be explained by clonal dissemination, as we detected closely related *E*. *coli* O6 isolates containing a class 1 integron with the *addA1* cassette, borne on a specific incF plasmid (F51:A-:B10). This *E*. *coli* variant, a previously described serotype involved in extraintestinal infections [39], occurred in patients hospitalized in six different hospitals spread across Norway.

Class 1 integrons are usually part of transposons enabling them to change location between different plasmids and between plasmids and the chromosome. Transposition into plasmids of different groups may facilitate a further spread of the integron structure in the bacterial populations. Mapping the flanking DNA of integrons in the isolates included in this study could give us valuable data on the commonness of the various transposons associated with integrons in the two groups of isolates. These data may also uncover some transposition events of importance for integron transfer to clinically important bacteria.

Global spread of ESBL producing *E*. *coli* during the last decade can to a large extent be explained by dissemination of successful pandemic *E*. *coli* clones like those belonging to the ST131 and ST405 genetic lineages [40,41]. Our findings demonstrate that antimicrobial resistance is also disseminated towards “older” antimicrobial agents such as streptomycin and sulphonamides via spread of specific clones. Until now, clonal spread of antimicrobial resistance in *E*. *coli* has mostly been investigated in highly multi-resistant and/or ESBL producing isolates.

One human isolate carried an integron with the *drfA1-aadA1* cassette array on an incF plasmid with the formula F2:A-:B-. This formula was originally assigned to plasmid R100 [24]. During the last decade, derivatives of this plasmid containing the ESBL gene *bla*_CTX-M-15_ have become widespread [24] and are probably the most frequent *bla*_CTX-M-15_ carrying plasmids circulating in *Enterobacteriaceae* [24]. Recent studies have also demonstrated plasmids with this formula containing other resistance determinants encoding resistance to aminoglycosides and fluoroquinolones [42]. Only three ESBL producers were identified in the present study, all of human origin. None of them contained integrons and were therefore not subjected to further analyses. The F2:A-:B- plasmid identified in this study is probably a derivative of the plasmid R100 without the gene encoding ESBL, thus demonstrating the plasmid´s ability to adapt, persist and acquire novel resistance genes over the years.

The results generated in this study could serve as baseline data from a time period predating the ESBL era in Scandinavia. It would be of interest to perform follow-up studies with a newer strain collection from our monitoring programs to investigate changes in the occurrence of integrons, changes in cassette composition, as well as changes in incF plasmids and their host strains. Such studies would provide valuable data concerning the evolution of antimicrobial resistance taking advantage of strain collections obtained through monitoring programs.

In this study we demonstrated some significant differences concerning integron prevalence and integron variants among the two categories of isolates investigated. However, closely related plasmids as vehicles for specific class 1 integrons in isolates from meat and from a human with septicaemia was found. The occurrence of similar multi-resistance plasmids in bacteria from a food source and from a human clinical sample highlights the possible role of meat as a source of resistance elements for pathogenic bacteria. However, further studies are needed to evaluate the extent of transmission and directions of transmission of resistance elements between the two *E*. *coli* populations.
